# Earlier Mother's Age at Menarche Predicts Rapid Infancy Growth and Childhood Obesity

**DOI:** 10.1371/journal.pmed.0040132

**Published:** 2007-04-24

**Authors:** Ken K Ong, Kate Northstone, Jonathan CK Wells, Carol Rubin, Andy R Ness, Jean Golding, David B Dunger

**Affiliations:** 1 Medical Research Council Epidemiology Unit, Cambridge, United Kingdom; 2 Department of Paediatrics, University of Cambridge, Cambridge, United Kingdom; 3 Department of Social Medicine, University of Bristol, United Kingdom; 4 Childhood Nutrition Research Centre, Institute of Child Health, London, United Kingdom; 5 National Center for Environmental Health, Centers for Disease Control and Prevention, Atlanta, United States of America; 6 Department of Community Based Medicine, University of Bristol, United Kingdom; London School of Hygiene and Tropical Medicine, United Kingdom

## Abstract

**Background:**

Early menarche tends to be preceded by rapid infancy weight gain and is associated with increased childhood and adult obesity risk. As age at menarche is a heritable trait, we hypothesised that age at menarche in the mother may in turn predict her children's early growth and obesity risk.

**Methods and Findings:**

We tested associations between mother's age at menarche, mother's adult body size and obesity risk, and her children's growth and obesity risk in 6,009 children from the UK population-based Avon Longitudinal Study of Parents and Children (ALSPAC) birth cohort who had growth and fat mass at age 9 y measured by dual-energy X-ray absorptiometry. A subgroup of 914 children also had detailed infancy and childhood growth data. In the mothers, earlier menarche was associated with shorter adult height (by 0.64 cm/y), increased weight (0.92 kg/y), and body mass index (BMI, 0.51 kg/m^2^/y; all *p* < 0.001). In contrast, in her children, earlier mother's menarche predicted taller height at 9 y (by 0.41 cm/y) and greater weight (0.80 kg/y), BMI (0.29 kg/m^2^/y), and fat mass index (0.22 kg/m^2^/year; all *p* < 0.001). Children in the earliest mother's menarche quintile (≤11 y) were more obese than the oldest quintile (≥15 y) (OR, 2.15, 95% CI 1.46 to 3.17; *p* < 0.001, adjusted for mother's education and BMI). In the subgroup, children in the earliest quintile showed faster gains in weight (*p* < 0.001) and height (*p* < 0.001) only from birth to 2 y, but not from 2 to 9 y (*p* = 0.3–0.8).

**Conclusions:**

Earlier age at menarche may be a transgenerational marker of a faster growth tempo, characterised by rapid weight gain and growth, particularly during infancy, and leading to taller childhood stature, but likely earlier maturation and therefore shorter adult stature. This growth pattern confers increased childhood and adult obesity risks.

## Abstract 2

Earlier age at menarche may be a transgenerational marker of faster growth, particularly during infancy, leading to taller childhood stature but earlier maturation and hence shorter adult stature.

## Editors' Summary

**Background.:**

Childhood obesity is a rapidly growing problem. Twenty-five years ago, overweight children were rare. Now, 155 million of the world's children are overweight and 30–45 million are obese. Overweight and obese children—those having a higher than average body mass index (BMI; weight divided by height squared) for their age and sex—are at increased risk of becoming obese adults. Such people are more likely to develop heart disease, diabetes, and other health problems than lean people. Many factors are involved in the burgeoning size of children. Parental obesity, for example, predisposes children to being overweight. In part, this is because parents influence the eating habits of their offspring and the amount of exercise they do. In addition, though, children inherit genetic factors from their parents that make them more likely to put on weight.

**Why Was This Study Done?:**

To prevent childhood obesity, health care professionals need ways to predict which infants are likely to become obese so that they can give parents advice on controlling their children's weight. In girls, early menarche (the start of menstruation) is associated with an increased risk of childhood and adult obesity and tends to be preceded by rapid weight gain in the first two years of life. Because age at menarche is inherited, the researchers in this study have investigated whether mothers' age at menarche predicts rapid growth in infancy and childhood obesity in their offspring using data from the Avon Longitudinal Study of Parents and Children (ALSPAC). In 1991–1992, this study recruited nearly 14,000 children born in Bristol, UK. Since then, the children have been regularly examined to investigate how their environment and genetic inheritance interact to affect their health.

**What Did the Researchers Do and Find?:**

The researchers measured the growth and fat mass of 6,009 children from ALSPAC at 9 years of age. For 914 of these children, the researchers had detailed data on their growth during infancy and early childhood. They then looked for any associations between the mother's age at menarche (as recalled during pregnancy), mother's adult body size, and the children's growth and obesity risk. In the mothers, earlier menarche was associated with shorter adult height and increased weight and BMI. In the children, those whose mothers had earlier menarche were taller and heavier than those whose mothers had a later menarche. They also had a higher BMI and more body fat. The children whose mothers had their first period before they were 11 were twice as likely to be obese as those whose mothers did not menstruate until they were 15 or older. Finally, for the children with detailed early growth data, those whose mothers had the earliest menarche had faster weight and height gains in the first two years of life (but not in the next seven years) than those whose mothers had the latest menarche.

**What Do These Findings Mean?:**

These findings indicate that earlier mother's menarche predicts a faster growth tempo (the speed at which an individual reaches their adult height) in their offspring, which is characterized by rapid weight and height gain during infancy. This faster growth tempo leads to taller childhood stature, earlier sexual maturity, and—because age at puberty determines adult height—shorter adult stature. An inherited growth pattern like this, the researchers write, confers an increased risk of childhood and adult obesity. As with all studies that look for associations between different measurements, these findings will be affected by the accuracy of the measurements—for example, how well the mothers recalled their age at menarche. Furthermore, because puberty, particularly in girls, is associated with an increase in body fat, a high BMI at age nine might indicate imminent puberty rather than a risk of long-standing obesity—further follow-up studies will clarify this point. Nevertheless, the current findings provide a new factor—earlier mother's menarche—that could help health care professionals identify which infants require early growth monitoring to avoid later obesity.

**Additional Information.:**

Please access these Web sites via the online version of this summary at http://dx.doi.org/10.1371/journal.pmed.0040132.

- The Avon Longitudinal Study of Parents and Children  has a description of the study and results to date
- The US Centers for Disease Control and Prevention provides information on overweight and obesity (in English and Spanish)
- US Department of Health and Human Services's program, Smallstep Kids, is an interactive site for children about healthy eating (in English and Spanish)
- The International Obesity Taskforce has information on obesity and its prevention
- The World Heart Federation's Global Prevention Alliance provides details of international efforts to halt the obesity epidemic and its associated chronic diseases
- The Child Growth Foundation has information on childhood growth and its measurement

## Introduction

Rising rates of obesity in young children, even at preschool ages [1], indicate that future preventative strategies against childhood and subsequent adult obesity will require the identification of their very early predictive factors. In adults, early age at menarche is a strong risk factor for increased risk of obesity in women [2–4]. However, the causal direction and mechanism behind this association are debatable, as girls with earlier menarche are more likely to be overweight even before the onset of puberty [5,6]. The link between earlier menarche and adult obesity may simply be due to their common association with previous overweight. Rapid infancy weight gain may be the common aetiological factor, as this early growth pattern is predictive for both earlier menarche and increased obesity risk [7,8]. The 1946 British National Birth Cohort showed that women with earlier menarche had rapid growth during the first 2 y of life followed by average growth rates between ages 2 through 7 y [7]. In contemporary birth cohort studies rapid infancy weight gain also predicts subsequent obesity risk in children and adults [8].

Age at menarche is a strongly heritable trait; in studies of twins 60%–80% of the variance is estimated to be genetic [9]. Despite recent identification of neuropeptide pathways involved in the activation of puberty, the factors that actually trigger this central process are still largely unclear [10]. There is a biological basis for a close link between nutritional status and the activation and maintenance of reproductive ability [11]. Factors that regulate infancy and early childhood growth, including genetic and epigenetic influences, could affect the timing of puberty [12]. We therefore hypothesised that mother's age at menarche may predict her offspring's early postnatal growth pattern and childhood obesity risk. We have examined this hypothesis in a large population-based birth cohort study: the Avon Longitudinal Study of Parents and Children (ALSPAC) [13].

## Methods

ALSPAC is a prospective study recruited from all pregnancies in three Bristol-based District Health Authorities with expected dates of delivery between April 1991 and December 1992, comprising 13,971 live births surviving to 1 y of age [13,14]. Birth weights were noted from hospital records and supine length was measured using a Harpenden neonatometer (Holtain Ltd, http://www.anthropometer.com) soon after birth (median 1 d, range 0–14 d) by the ALSPAC study team. Gestation was estimated using the date of last menstrual period and confirmed by antenatal ultrasound reports; in cases of discrepancy the data were reviewed by a single experienced clinician. Mother's parity and smoking during pregnancy were recorded by a questionnaire completed during pregnancy. The mother's highest educational achievement was recorded by questionnaire as an indicator of her socioeconomic status. Ethical approval was obtained from the ALSPAC and the local research ethics committees. Signed consent for anthropometry was obtained from a parent or guardian and verbal assent was obtained from the children.

### Age at Menarche

Mother's age at menarche was recorded in completed years by recall in response to a questionnaire administered during pregnancy. Mother's prepregnancy height and weight were also recorded by questionnaire. Age at menarche in the daughters was assessed by status quo (i.e., had menstrual periods started or not) in a questionnaire completed at age 10 y.

### Assessment at Age 9 Years

At age 9.9 (± 0.33) y, all offspring were invited to attend a research clinic for a 3-h assessment, which included a session of anthropometric measurements. Height was measured with shoes and socks removed using a Harpenden stadiometer (Holtain Ltd). Weight was measured using a Tanita TBF 305 body fat analyser and weighing scales (Tanita UK Ltd, http://www.tanita.co.uk). Whole-body fat mass was measured using dual-energy X-ray absorptiometry (DXA; Lunar Prodigy, GE Medical Systems, http://www.gehealthcare.com/) and performed by trained fieldworkers, The scans were visually inspected and realigned where necessary. We performed repeated DXA measurements for 122 children on the same day, and the repeatability coefficient (twice the standard deviation of the difference between measurement occasions) [15] for body fat mass was 0.5 kg.

### Children in Focus Subcohort

From the whole ALSPAC cohort, a 10% “Children in Focus” subcohort (1,335 full-term singleton infants) was randomly selected from the last 6 months of recruitment for more detailed and regular measurements of infant and childhood growth on up to ten occasions until 5 y (at 4, 8, 12, 18, 25, 31, 37, 43, 49, and 61 mo), and the whole cohort were invited for measurements at 7 and 9 y [16,17]. Complete data for this analysis were selected from measurements at birth and 2, 5, 7, and 9 y. Weight (measured on Seca 724 or 835 scales) and standing height (using Leicester height measures, http://www.childgrowthfoundation.org/) were measured at follow-up visits to the research clinic. Breast-feeding was recorded as current status at age 4 mo (including up to one formula feed per day), and age at introduction of solids was recorded by questionnaire.

### Calculations

As measures of weight-for-height, ponderal index at birth was calculated as birth weight/birth length^3^ (kg/m^3^), and body mass index (BMI) was calculated as weight/height^2^ (kg/m^2^) in the children at age 9 y and in the mothers pre-pregnancy. Fat mass was corrected for height by calculating the fat mass index fat mass / height^2^ (kg/m^2^) [18]. Truncal fat index was similarly calculated. Obesity was defined in mothers as BMI > 30 kg/m^2^, and in children as BMI > 97th percentile for sex and age by comparison with the UK 1990 growth reference [19]; use of different cutoffs produced very similar risk scores (unpublished data).

In the Children in Focus subcohort, all weight and length measurements were converted to sex- and age-independent standard deviation (SD) scores in each participant by comparison with the UK 1990 growth reference [19]. Weight gain and growth were calculated as changes in SD score over time (between 0–2 y and 2–9 y).

### Statistics

Mother's age at menarche showed a normal distribution (mean ± SD = 12.8 ± 1.5 y), and values were divided into approximate quintiles for analysis and display. BMI, fat mass index, and truncal fat index showed skewed distributions; analyses were therefore based on log-transformed data, and the data in Table 1 represent geometric means. Multiple linear regression models were performed to test the associations between quintiles of mother's age at menarche (adjusted for sex, age, and mother's educational achievement) and various outcome measures of mother's body size and children's body size, body composition, and growth. Models with outcome measures of infant (0–2 y) growth were further adjusted for gestational age, parity, and breast-feeding status at 4 mo. Statistical comparisons of obesity risk between the earliest and the oldest quintiles of mother's menarche were performed by multivariate logistic regression, resulting in the estimation of adjusted odds ratios (OR) and 95 percent confidence intervals (CIs). For childhood obesity risk, mother's BMI was entered into the model as a covariable, because mother's prepregnancy BMI was positively related to her child's BMI at 9 y (*r* = 0.31, *p* < 0.001) [16].

**Table 1:**
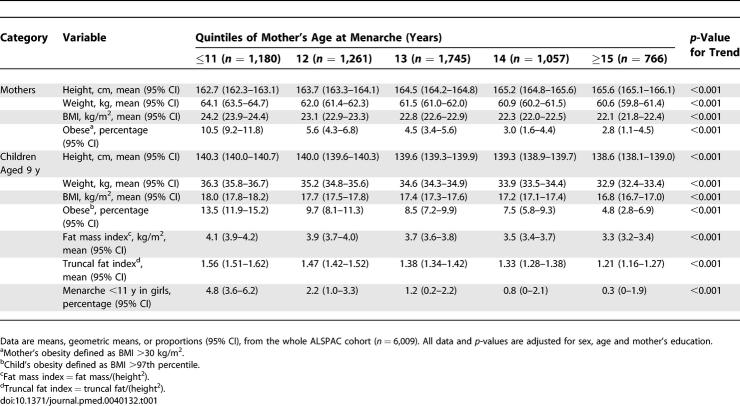
Body Size and Obesity Risk of Mothers and Children at 9y by Quintiles of Mother's Age at Menarche

## Results

In the mothers, early age at menarche was linearly associated with reduced adult height (by 0.64 cm/y; *p* < 0.001), but increased adult body weight (0.92 kg/y; *p* < 0.001) and BMI (0.51 kg/m^2^/y; *p* < 0.001; Table 1). Compared with the oldest menarche quintile (≥15 y old), those mothers in the earliest menarche quintile (≤11 y) had a 5-fold increased risk of obesity (OR 5.11, 95% CI 3.41–7.67; *p* < 0.001, adjusted for mother's age and education).

**Figure 1:**
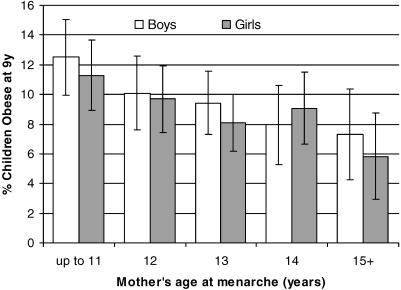
Prevalence of Obesity in Children at Age 9 y, by Mother's Age at Menarche Data are proportions (± 95% CI) of children with obesity (BMI >97th percentile according to the UK 1990 growth reference [19]) in each quintile of mother's menarche from the whole ALSPAC cohort (boys: *p*-value for trend = 0.003, *n* = 2,961; girls: *p*-value for trend = 0.006, *n* = 3,048), adjusted for age, mother's education, and also for mother's BMI.

Children of those mothers with earlier menarche had taller heights at age 9 y (by 0.41 cm/y; *p* < 0.001), increased body weight (0.80 kg/y; *p* < 0.001) and BMI (0.29 kg/m^2^/y; *p* < 0.001; Table 1). Most of the gain in BMI was attributable to greater fat mass index (0.22 kg/m^2^/y; *p* < 0.001) rather than lean mass index (0.05 kg/m^2^/y; *p* < 0.001). Daughters of mothers with earlier menarche were themselves more likely to report early menarche (before age 11 y; *p* < 0.001; Table 1). Compared with children of mothers in the oldest menarche quintile (≥15 y), children of mothers in the earliest menarche quintile (≤11 y) had a nearly 3-fold increased risk of obesity (OR 2.91, 95% CI 2.02–4.19; *p* < 0.001, adjusted for sex, age, and mother's education). The risk of obesity was similar in boys and girls (*p*-value for interaction = 0.9; Figure 1), and it was only partially attenuated by further adjustment for mother's BMI (OR 2.15, 95% CI 1.46–3.17; *p* < 0.001; Figure 1).

In addition, further adjustments for mother's BMI only partially attenuated the other associations seen between earlier mother's menarche and larger size of her children at 9 y: height (0.35 cm/y; *p* < 0.001), body weight (0.54 kg/y; *p* < 0.001), BMI (0.18 kg/m^2^/y; *p* < 0.001), fat mass index (0.14 kg/m^2^/y; *p* < 0.001), and lean mass index (0.03 kg/m^2^/y; *p* = 0.002).

**Figure 2:**
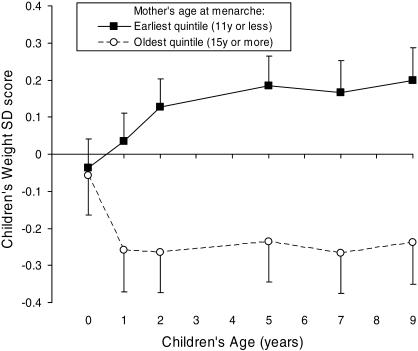
Contrasting Early Postnatal Growth Patterns in Offspring of Mothers with Earlier or Later Menarche Unadjusted weight SD scores are shown in children grouped by extreme quintiles of their mother's age at menarche. Data are means ± standard error from the Children in Focus subgroup (*n* = 914).

In a subcohort of the ALSPAC study in whom detailed early postnatal growth data were collected (*n* = 914), mother's age at menarche was inversely related to her offspring's infancy weight gain (*p* < 0.001) and height gain (*p* < 0.001) between birth and 2 y (adjusted for sex, gestational age, parity, mother's BMI, and breast-feeding status at age 4 mo [Table 2]). These associations were similar in boys and girls (*p*-value for interaction = 0.8), and in breast-fed and formula milk–fed infants (*p*-interaction = 0.9). However, mother's age at menarche was unrelated to her offspring's size at birth or later childhood growth rates (between 2–9 y) (Table 2). Figure 2 shows the contrasting early growth patterns between children in the earliest and oldest quintiles for mother's age at menarche.

**Table 2:**
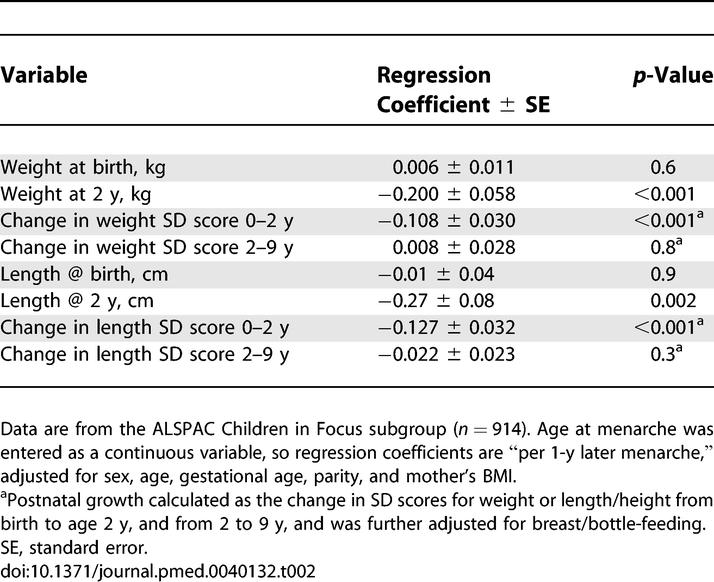
Association between Mother's Age at Menarche and Her Children's Size at Birth and Postnatal Growth

## Discussion

Our data from a large, representative UK birth cohort show that earlier mother's age at menarche is a marker not only for her own obesity risk, but is also associated with faster weight gain and growth during infancy, and increased fat mass and obesity risk in her children. These findings suggest that the consistent association between rapid infancy weight gain and later obesity risk [8] has an inherited or transgenerational component.

We did not have data on the mother's own growth rates during infancy. However, previous studies have reported a strong association between infancy growth and the timing of sexual maturation in males and females [7,20,21]. Our observation that mother's age at menarche in turn predicts her offspring's infancy growth rate and daughter's menarche provide some important insights into the transgenerational influences on childhood growth.

“Growth tempo” was coined from the musical term to indicate how fast an individual reaches their full adult height [22]. It has long been recognised that some children are “rapid maturers,” who are larger than their peers only transiently during childhood [23,24]. The contrasting associations we observed between earlier menarche and shorter adult height in the mothers, yet with taller offspring height during childhood indicate that many individuals who show rapid infancy growth and are taller than average during childhood are likely destined to be relatively shorter as adults. Whether age at puberty influences adult height has been debated [25]; however, recent large international studies support our current finding [26], and it is likely that earlier studies were underpowered. Recognition of such potentially large differences in growth tempo may help to clarify the often variable life-course associations reported between childhood growth and adult disease risks [27]. Our findings suggest that growth tempo is a heritable trait that becomes established very early on, within the first two years of life.

This intergenerational link between age at menarche and infancy weight gain might be explained by genetic factors transmitted to the offspring from the mother. Both age at menarche and infancy growth rates show high heritability in studies comparing monozygous with dizygous twins [9,22,28]. Specific genes for either trait have yet to be identified, but our findings would support a common genetic pathway associated with both infancy growth and the tempo of childhood growth and puberty. Potential candidates are genes that regulate early appetite and satiety [29]. Genetic factors that influence sex hormone activity might also regulate both puberty and infant growth, as there is a mini-activation of central and peripheral sex hormones during infancy [30,31]. We have no reliable information on father's pubertal development as, in contrast to menarche in females, there are few robust self-reportable markers of puberty in males. Mother's menarche had a similar influence on her sons and daughters, and it is possible that rapid puberty in fathers might also in turn influence offspring growth.

Recent research has also emphasised the importance of nongenetic modes of biological heritability, including transgenerational hormonal programming [32], epigenetics [33], and behaviour [34]. Nutrition has a major influence on infancy weight gain and growth, and dietary intake depends on both parental choices and the expression of genetic factors in the infant [35,36]. Variations in nutrition during infancy are associated with subsequent obesity risk [37] and timing of menarche in humans [38], and have been shown to program long-term gene methylation and expression in mice [39]. Our finding that the faster growth rate of those destined to reach menarche earlier occurs only during the first two years of life may indicate that the underlying mechanism has a programmed or epigenetic component related to early nutritional intake. Growth is energetically expensive in early life, and generally in mammals, lactation is considerably more costly in terms of energy than gestation [40]. A mother with larger energy reserves or a history of rapid development could somehow signal to her offspring to set up a rapid postnatal growth trajectory, possibly through programming or epigenetic modulation of genes relating to appetite and growth, and thereby steer her offspring toward earlier maturation.

In our study, the association between mother's menarche and offspring obesity could remain open to potential residual confounding by the mother's BMI, which was assessed by self-reported height and weight and therefore likely to be underestimated, or by other unmeasured social or behavioural factors related to the mother's obesity. However, the mother's BMI was unrelated to offspring infant weight gain (correlation: *r* = 0.03) [16], and therefore the associations between mother's age at menarche and her infant's weight gain and growth are unlikely to be confounded by mother's BMI. Age at menarche in the mothers was reported by recall many years after the event. Other studies have shown very good correlations (*r* = 0.67–0.79) between age at menarche by recall during middle age and the original childhood data [41,42], and the validity of our data in the mothers is supported by the expected strong associations with mother's BMI, and with early menarche in their daughters.

In conclusion, earlier age at menarche may indicate a transgenerational influence toward a faster tempo of childhood growth, which is transmitted from the mother to her offspring. The trait is manifested in early postnatal life by promoting weight gain and growth during infancy, taller childhood but shorter adult stature due to earlier completion of growth, and increased childhood and adult obesity risks. Earlier mother's menarche and maternal obesity independently predispose to childhood obesity, and both factors could be used to indicate which infants might require closer early growth monitoring. Finally, understanding the genetic, epigenetic, or behavioural factors that underlie this process will identify processes that regulate both the timing of puberty and the risk of childhood-onset obesity.

## Abbreviations

ALSPAC: The Avon Longitudinal Study of Parents and Children
BMI: body mass index
CI: confidence interval
DXA: dual-energy X-ray absorptiometry
OR: adjusted odds ratio
SD: standard deviation
